# Acupuncture for acute stroke: study protocol for a multicenter, randomized, controlled trial

**DOI:** 10.1186/1745-6215-15-214

**Published:** 2014-06-08

**Authors:** Lifang Chen, Jianqiao Fang, Ruijie Ma, Ronen Froym, Xudong Gu, Jianhua Li, Lina Chen, Shouyu Xu, Conghua Ji

**Affiliations:** 1 Department of Acupuncture, The Third Affiliated Hospital of Zhejiang Chinese Medical University, No. 219 Moganshan Road, XiHu District, Hangzhou, Zhejiang Province 310005, China; 2 The Third Clinical Medical College of Zhejiang Chinese Medical University, No. 548 Binwen Road, Binjiang District, Hangzhou, Zhejiang Province 310053, China; 3 Department of Rehabilitation, The Second Hospital of Jiaxing, No. 1518 North HuanCheng Road, JiaXing, Zhejiang Province 314000, China; 4 Department of Rehabilitation, Sir Run Run Shaw Hospital (ZheJiang University School of Medicine), No. 3 East Qingchun Road, Hangzhou, Zhejiang Province 310016, China; 5 Department of Rehabilitation, The First People’s Hospital of Hangzhou, No. 261, HuanSha Road, Hangzhou, Zhejiang Province 310006, China; 6 Department of Rehabilitation, The Third Affiliated Hospital of Zhejiang Chinese Medical University, No. 219 Moganshan Road, XiHu District, Hangzhou, Zhejiang Province 310005, China; 7 The Clinical Research Institute of Zhejiang Provincial Hospital of TCM, No. 54 Youdian Road, Hangzhou, Zhejiang Province 310006, China

**Keywords:** Acupuncture, Stroke, Multicenter RCT

## Abstract

**Background:**

Acupuncture has been widely used as a treatment for stroke in China for more than 3,000 years. However, previous research has not yet shown that acupuncture is effective as a stroke treatment. We report a protocol for a multicenter, randomized, controlled, and outcome assessor-blind trial to evaluate the efficacy and safety of acupuncture on acute ischemic stroke.

**Methods/Design:**

In a prospective trial involving three hospitals in the Zhejiang Province (China) 250 patients with a recent (less than 1 week previous) episode of ischemic stroke will be included. Patients will be randomized into two groups: an acupuncture group given scalp acupuncture and electroacupuncture, and a control group given no acupuncture. Eighteen treatment sessions will be performed over a three-week period. The primary outcome will be measured by changes in the National Institutes of Health Stroke Scale score at the one, three, and four-week follow-up. Secondary outcome measures will be: 1) the Fugl-Meyer assessment scale for motor function; 2) the mini-mental state examination and Montreal cognitive assessment for cognitive function; 3) the video-fluoroscopic swallowing study for swallowing ability; and 4) the incidence of adverse events.

**Discussion:**

This trial is expected to clarify whether or not acupuncture is effective for acute stroke. It will also show if acupuncture can improve motor, cognitive, or swallowing function.

**Trial registration:**

Chinese Clinical Trial Registry ChiCTR-TRC-12001971.

## Background

Stroke is a major cause of death and disability worldwide [1-3]. It is also the leading cause of death and long-term disability in China [4,5], where a rising incidence of stroke has created a serious public health problem [6,7]. Despite a considerable amount of research on treatment for stroke there is still no single intervention that clearly and definitely contributes to stroke recovery. However, there is no doubt that the treatment of stroke should be combined with multiple disciplines such as neurology, rehabilitation medicine, and traditional medicine. Acupuncture has been used in traditional Chinese medicine for more than 3,000 years as a treatment for many diseases and is especially well accepted in China for post-stroke rehabilitation [8]. Because stroke is one of the most common uses of acupuncture therapy, many Chinese acupuncture departments have set up special wards to treat stroke patients to maximize the promotion of recovery. It has been shown that acupuncture can cause multiple biological responses including circulatory and biochemical effects [9,10]. As a therapeutic intervention acupuncture is used to improve motor, sensation, speech, swallowing, cognitive, and other neurological functions in patients with stroke [11,12]. Scalp acupuncture seems to improve the neurological deficit score and clinical effective rate when compared with Western conventional medicines alone [13], and it produces positive but limited effectiveness as an adjunct treatment to conventional care [14]. Electroacupuncture appears to be beneficial for the motor function recovery of patients with acute ischemic stroke and is generally safe [15]. However, Cochrane reviews have shown that while acupuncture appears to be safe, there is no clear evidence of benefit [16]. Therefore, there is insufficient data on the routine usage of acupuncture in acute stroke. More rigorously designed, large, multicenter, randomized trials are needed to further assess the effectiveness of acupuncture on stroke rehabilitation.

## Methods/Design

### Study design

**Figure 1:**
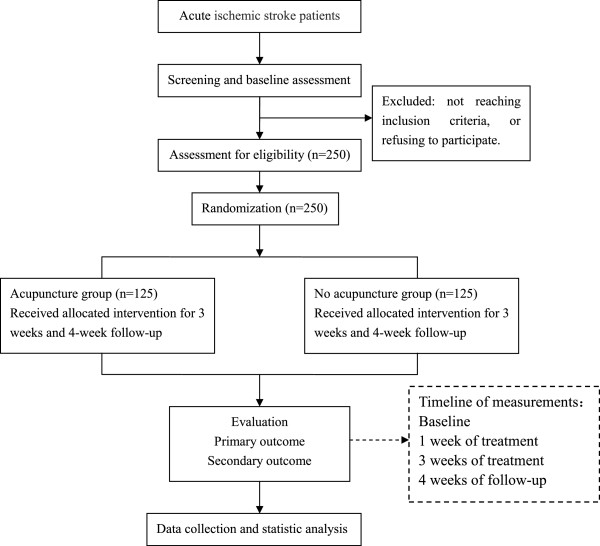
Flow diagram of study design.

The study is a prospective, multicenter, outcome assessor-blinded, randomized controlled clinical trial carried out in three inpatient stroke rehabilitation units. After the inclusion and consent of the Institutional Review Board, participants will be randomized to either the control (no acupuncture) or the acupuncture group. The control group will receive conventional stroke rehabilitation care (including physical and occupational therapy, swallowing training for dysphagia, and/or cognitive training for cognitive impairment; six days per week) during the inpatient stay. The acupuncture group will receive conventional stroke rehabilitation care and 30 additional minutes of acupuncture therapy six days per week for three weeks (18 total acupuncture sessions) during the inpatient stay. The trial design is summarized in Figure 1.

### Participant recruitment

Participants will be recruited in three large hospitals (The Second Hospital of Jiaxing City, Sir Run Run Shaw Hospital, and The First People’s Hospital of Hangzhou) in Zhejiang Province, China. Our study will be propagated by local newspapers, health-related TV programs, the internet, and posters in communities and hospitals. Prospective participants will be asked to talk face-to-face with study coordinators to discuss the study and provide information regarding eligibility criteria. If patients are eligible and interested in participating they will be invited for a series of rehabilitation assessments after diagnosis by neurologists. Two hundred and fifty consecutive patients admitted to the three hospitals because of acute ischemic stroke will be included in the study. When their informed consent has been obtained, patients will be randomized into two groups with different treatments.

### Inclusion criteria

We will choose patients aged between 35 and 80-years-old who are hospitalized with acute ischemic stroke according to the criteria of both Western medicine and traditional Chinese medicine. The stroke onset must be between two to seven days beforehand, with a National Institutes of Health Stroke Scale (NIHSS) score of between 4 and 24. The stroke should be the first incidence or patients can have a history of stroke but without disability (modified Rankin Scale, mRS score ≤1).

### Exclusion criteria

Patients who received thrombolytic therapy or who participated in other clinical trials within last three months are not included. Patients who suffered from serious heart,liver,kidney related diseases, blood coagulation dysfunction, or severe mental disorders will not be included. Women who are pregnant or breast-feeding and patients with a severe cognitive impairment are also excluded.

### Ethical considerations

Each ethics committee of the Third Affiliated Hospital of Zhejiang Chinese Medical University, The Second Hospital of Jiaxing City, and Sir Run Run Shaw Hospital, together with The First People’s Hospital of Hangzhou, all approved the study. The purpose, nature, and potential risks of the experiments were fully explained to the patients and their families. All patients gave their written informed consent before participating in the study.

### Randomization and blinding

Randomization to acupuncture or no acupuncture will be computer-generated by independent research staff using software. The treatment codes are placed in sealed opaque envelopes which will be distributed by research nurses who will be trained before the trial and will not participate in treatment or nursing. Patients in this research will be told that they will receive routine rehabilitation treatment with or without acupuncture. It is impossible to blind the acupuncturists because they must be trained to perform the acupuncture according to the research plan. A special rehabilitation doctor from each center will be assigned to be the assessor who is blinded to the patient treatment group and does not participate in the treatment of subjects. All study personnel including the rehabilitation therapists, outcome assessors, and data analysts will be blinded to group assignments.

### Interventions and comparison

The study is a randomized clinical trial carried out in three inpatient rehabilitation departments of three hospitals. Participants will be randomized to either the control or the acupuncture group. Both groups will receive conventional stroke rehabilitation care, which begins as soon as the diagnosis of stroke is established and life-threatening problems are under control. The basic rehabilitation care includes normal limb posture, passive motion with hemiplegia side, bedside rehabilitation (Bobath technique, overturning movement, bridge movement), and/or neuromuscular electrical stimulation, swallow training for dysphagia, and/or cognitive training for cognitive impairment. The rehabilitation program (including physiotherapy and occupational therapy for two hours per day, six days per week) of each participant will be developed by the rehabilitation team according to the investigator’s brochure. The acupuncture group will receive 30 additional minutes of acupuncture therapy for six days per week for three weeks (18 total sessions) during the inpatient stay.

#### Acupuncture group

The acupuncture protocol is designed by a professor from Zhejiang Chinese Medical University who has more than 20 years of practice experience, and will be performed by three doctors from the acupuncture department who have a master’s degree with more than five years of clinical experience. They will be trained together and use the same techniques. The treatment will be started in both groups after randomization. The acupuncture protocol is split according to the area of treatment.

The parameters for scalp acupuncture are set as follows: two to three needles penetrating through the top midline, the motor region, and the sensory region of the lesion side. The parameters for the affected side of the body are as follows: LI15 (Jian Yu), LI11 (Qu Chi), LI10 (Shou San Li), SJ5 (Wai Guan), LI4 (He Gu) for upper extremities; St34 (Liang Qiu), St36 (Zu San Li), GB34 (Yang Ling Quan), Sp6 (San Yin Jiao), St40 (Feng Long), St41 (Jie Xi), and Liv3 (Tai Chong) for lower limbs. The parameters for acupoint modification are set as follows: For dysphagia, GB20 (Feng Chi), EX-HN14 (Yi Ming), BL10 (Tian Zhu), DU16 (Feng Fu), Gong Xue (1 cun below GB20), RN23 (Lian Quan) will be added. For mild cognitive impairment, DU20 (Bai Hui), DU24 (Shen Ting), GB13 (Ben Shen), EX-HN1 (Si Shen Cong) will be added.

Manual stimulation will be applied to the body acupoints until the patients experience De qi (get Qi). Patients experience De qi as multiple unique sensations at the needle site itself and around the site of needle manipulation, including soreness, aching, numbness, tingling, and even warmth [17,18]. The parameters for electroacupuncture for LI15 (Jian Yu) and LI11 (Qu Chi), St36 (Zu San Li) and Sp6 (San Yin Jiao) are set as follows: intermittent wave, low-frequency (2 Hz); the intensity is within the scope that patients can tolerate. Huatuo brand needles (size 0.25 mm × 40 mm) were manufactured by Suzhou Medical Appliance in Suzhou, Jiangsu Province China, with GB6805-2 Electro-Acu Stimulators (Huayi Medical Supply & Equipment Co. Ltd, Shanghai, China) used.

#### No acupuncture treatment group

Patients in the control group will receive conventional stroke rehabilitation care, which is the same as the acupuncture group. However, no acupuncture treatments will be given during the study period. They will be assessed at each visit.

### Outcome assessment

The patients will be carefully examined at baseline, re-examined after one week of treatment, and again after the last treatment (three weeks after first acupuncture). There will be a four-week follow-up after completion of the acupuncture treatment.

#### Baseline assessments

Baseline assessments will be conducted before randomization, including gender and age of patients, clinical syndrome (left or right hemiplegia), diseased location and size, NIHSS and Fugl-Meyer assessment (FMA) scores, cognitive impairment based on the Montreal cognitive assessment (MoCA), and swallowing disorder according to the standardized swallowing assessment.

#### Primary outcome measurement

The primary outcome measurement in the study is the change in NIHSS scores for neurological deficit evaluation. NIHSS is a standardized stroke severity scale used to describe neurological deficits in stroke patients and it strongly predicts the likelihood of a patient’s recovery after stroke. The NIHSS comprises tests of 11 items including the level of consciousness, best gaze, visual field, facial palsy, motor arm, motor leg, limb ataxia, sensory ability, best language, dysphagia, and neglect. Total scores range from 0 to 42, with scores above 25 indicating very severe neurological impairment, scores of 5 to 24 suggesting moderately severe to severe impairment, and scores below 5 indicating mild impairment [19]. Assessments will be conducted at baseline and at one, three and four-weeks follow-up.

#### Secondary outcome measures

**Fugl-Meyer Assessment (FMA) scale for motor function** The FMA was developed as the first quantitative evaluative instrument for measuring sensorimotor stroke recovery, which includes an assessment of the upper extremity (UE, 66 points) and lower extremity (LE, 34 points). The motor domain has well-established reliability and validity as an indicator of motor impairment severity across different stroke recovery time points [20].

**The mini-mental state examination (MMSE) and Montreal cognitive assessment (MoCA) for cognitive function** The MMSE, first introduced by Folstein and colleagues in 1975 [21], has become a standard tool for cognitive assessment in the clinical setting. It is a 10-minute bedside measure of impaired thinking in undeveloped, uneducated, diseased, or very old populations. The summed score of the individual items indicates the current severity of cognitive impairment [22]. However, there are many difficulties in detecting early dementia with the MMSE. Most individuals meet clinical criteria for mild cognitive impairment (MCI), but have scores above 26 on the MMSE, which is within the range for normal elderly individuals. To address this problem the MoCA was developed as a tool to screen patients who present with mild cognitive complaints and usually perform in the normal range on the MMSE [23]. The MoCA is used as a screening tool for MCI in baseline assessment in our study.

**Video-fluoroscopic swallowing study (VFSS) for swallowing ability evaluation** The functional dysphagia scale is based on the VFSS in stroke patients, which is a sensitive and specific method for quantifying the severity of dysphagia [24,25].

**Incidence of adverse events** The subjects will be requested to voluntarily report information about adverse events. All adverse events happening during the trial will be recorded, such as pallor, sweating or dizziness, fainting during acupuncture treatment, bleeding, local hematoma, unbearable prickling, retained needle after treatment, and continuous severe pain more than one hour after acupuncture. The researcher will confirm the occurrence of adverse events and record all details such as the date of occurrence, time, degree, measurement related to the treatment, and causal relationship with the treatment. Serious adverse events will be reported to the principal investigator immediately.

### Quality control

The trial protocol has been reviewed and revised by experts on acupuncture, neurology, rehabilitation, statistics, and methodology several times. Before the trial, all staff are required to attend a series of training sessions. These sessions will ensure that the personnel involved fully understand the research protocol and standard operating procedures for the study. During the trial supervisors will check on case reports and acupuncture operation twice per month. The research team will meet regularly (once every three months) throughout the trial to discuss progress, including recruitment, withdrawals, treatment compliance, and adverse events. Economic compensation and health education are also considered as methods for improving compliance. A Data and Safety Monitoring Board was set up on 10 May 2012 to monitor the performance and safety of the trial.

### Sample size calculation

The primary efficacy parameter is the change in NIHSS scores from baseline to the end of treatment after three weeks. According to our preliminary test and previous study [26], the decrease in NIHSS score was conservatively estimated to be 5 in the acupuncture group and 3 in the control group, with a standard deviation of 5.0. A two-sided 5% significance level and 80% power were considered, and the following equation was used:

(1) n=2za2+zβ2σ2Δ2

Approximately 100 participants in each group were calculated to be required. Estimating a 20% dropout rate, each group will require 125 initial participants.

### Statistical analysis

All data will be analyzed by a blinded statistician using the SPSS(Statistical Product and Service Solutions) statistical package program (version 17.0, SPSS Inc., Chicago, Illinois, United States) at a separate location from the clinical research institute of Zhejiang Provincial Hospital of Chinese medicine. Baseline data will be collected and compared first. All patients randomized to each group are included in the analysis and the data analysis will be conducted using two-sided significance tests at the 5% significance level. The analysis of swallowing disorder and cognitive impairment will be made on the defined population of corresponding dysfunction in the baseline assessment. Different statistics are presented differently; for example, continuous variables will be expressed as mean ± SD, *t*-test will be used for comparisons of two independent samples, and non-parametric tests will be used for variables that do not follow a normal distribution. A *P* value of less than 0.05 (α value of 0.05) is considered to indicate statistical significance. A safety analysis will be performed by analyzing the frequency of adverse events suspected as related to the treatment. The data for adverse events are collected through the symptoms reported by the patients, and observations by a researcher at every visit.

## Discussion

Acupuncture is widely accepted by Chinese people and it is increasingly requested by patients and their relatives in Western countries. Stroke is one of the most common diseases for which acupuncture treatment is recommended, according to the World Health Organization [27]. However, Cochrane reviews have shown that although acupuncture appeared to be safe, there is no clear evidence of benefit. The number of patients in existing trials is too small to be certain whether acupuncture is effective for the treatment of acute ischemic stroke. Larger, methodologically sound trials are required [16,28].

For clinical trials blinding is difficult for acupuncture so real randomized placebo-controlled trials seem impossible. In Western medical hospitals in China, acupuncture is used less for stroke compared with Chinese medical hospitals, so setting up a no acupuncture control is feasible. We will conduct our trial in three Western medicine hospitals to ensure a sufficient source of subjects and compliance of the control group. Because many stroke patients will ask for traditional Chinese medicine and acupuncture treatment during the recovery period, we have set the follow-up period as only four weeks.

Under strict quality control, this study could potentially confirm whether or not acupuncture (including scalp acupuncture and electroacupuncture) is an effective adjunct to the standard rehabilitation therapy for acute stroke. Our study may also confirm if acupuncture can be effective in promoting the recovery of motor function and is beneficial to swallowing disorder and cognitive impairment.

## Trial status

Participant enrollment is started from 1 March 2012. Enrollment and trial completion are expected to be finished by the end of June 2014.

## Abbreviations

FMA: Fugl-Meyer Assessment; MCI: Mild Cognitive Impairment; MMSE: Mini-Mental State Examination; MoCA: Montreal Cognitive Assessment; NIHSS: National Institutes of Health Stroke Scale; VFSS: Video-fluoroscopic Swallowing Study; SPSS: Statistical Product and Service Solutions; mRS: modified Rankin Scale.

## Competing interests

The authors declare that they have no competing interests.

## Authors’ contributions

LFC, JQF, RJM, RF, SYX and CHJ participated in the conception and design of the trial, planning the analysis of the data and drafting the manuscript. XDG, JHL, LNC participated in data collection and are in charge of recruitment and treatment of patients in each center. All the authors discussed, revised and approved the final manuscript.
